# Untangling the Effects of Hydraulic Design on Opportunistic Pathogen Growth Potential with an at-Scale Plumbing Rig

**DOI:** 10.1021/acsestwater.4c00812

**Published:** 2025-01-03

**Authors:** Sarah Busch, Tolulope O. Odimayomi, William J. Rhoads, Amy Pruden, Marc A. Edwards

**Affiliations:** Via Department of Civil and Environmental Engineering, Virginia Tech, Blacksburg, Virginia 24061, United States

**Keywords:** water retention time, water age, stagnation, flow, flow rate, flow velocity, *Legionella*, *Mycobacterium*, microbial regrowth

## Abstract

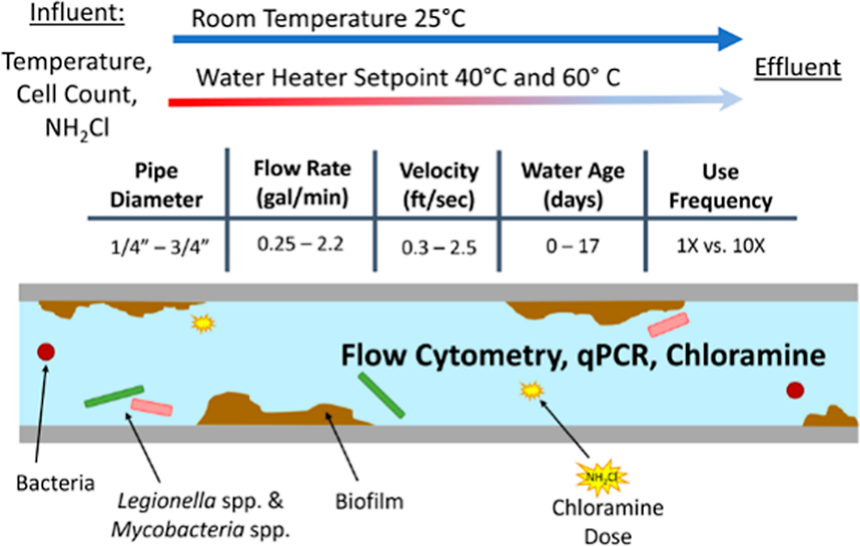

A building plumbing rig experiment simultaneously examined how water temperature (cold/hot lines), influent disinfectant residual (0–1 mg/L chloramine), flow rate (0.5–2.2 gpm), and water retention time (WRT) of 0–17-days impacted water quality at the point of use. In cold water lines with no disinfectant, WRT was a key driver of bacterial growth, with total cell counts (TCC) in the water increasing by up to 20× relative to influent water at 6.7-days WRT. A chloramine residual in cold influent water suppressed the maximum TCC by about 50%, even after the residual was no longer measurable. When the water heater set point was warm (40 °C) with minimal or 1 mg/L Cl_2_ chloramine, the majority of microbial growth occurred in the tank (WRT = 3 days). However, at a heater set point of 60 °C with 1 mg/L as Cl_2,_ growth was completely repressed in the tank, shifting growth to the distal pipes. *Legionella* spp. gene copies measured in cold bulk water increased with WRT, but not flow velocity. In hot water biofilms, *Legionella* spp. gene copies were highest at low WRT and high flow velocities. *Mycobacterium* spp. gene copies in hot water biofilms escalated after chloramines were introduced and were positively correlated to water velocity.

## Abstract 2

An at-scale pipe rig revealed that the location of optimal growth niches for *Legionella* spp., *Mycobacterium* spp., and total bacteria is dependent on water age, water heater set point, and influent disinfectant level.

## Introduction

Potable water conservation efforts have focused intensively on reducing the flow rate of water fixtures,^1^ increasing water retention times (WRTs) within premise plumbing, and decreasing water velocities in pipes unless pipe diameter, water storage volumes, or flushing practices are altered.^2,3^ Elevated building WRT (i.e., the time water is held in plumbing while traveling from the point-of-entry to a tap where it is drawn) is associated with numerous water quality problems, including reduced efficacy of corrosion control, degraded aesthetics (i.e., tastes, odors, and discoloration), and lower levels of secondary disinfectant residuals, which can increase microbial growth.^3−10^ Low flow rates are also less effective at sweeping out sediment and organic matter, which can accumulate in pipes and further exacerbate decay of disinfectant residual and microbial growth.^11,12^

Elevated WRT has also been associated with higher levels of opportunistic pathogens (OPs) in some studies.^7,9,13−16^ OPs can establish in building plumbing growth niches and are recognized as the primary source of waterborne disease in many developed countries.^17−20^ Although numerous studies have reported increased OP colonization with low flow rates relative to conventional faucets,^21−27^ controlled experiments are needed to confirm such associations and elucidate the controlling mechanisms.

A complex array of factors is expected to influence the net effect of the flow rate on OP growth. For instance, low flow rates (e.g., laminar) might increase OP growth relative to higher flow by allowing sediment to accumulate or failing to effectively deliver disinfecting chemicals or heat to biofilms. However, when biofilms are nutrient-limited, the lower flow rate and frequency might inhibit OP growth.^6,28−31^

Here, we developed a novel experimental pipe rig apparatus that allows for at-scale replication of multiple building plumbing scenarios. Experiments tested effects of illustrative extremes of WRT, as controlled by the water flow rate, pipe diameter, pipe length, and differing water use scenarios, on OP growth potential and overall microbial and chemical water quality at sampling taps.

## Methods

### At-Scale Pipe Rig Apparatus

**Figure 1:**
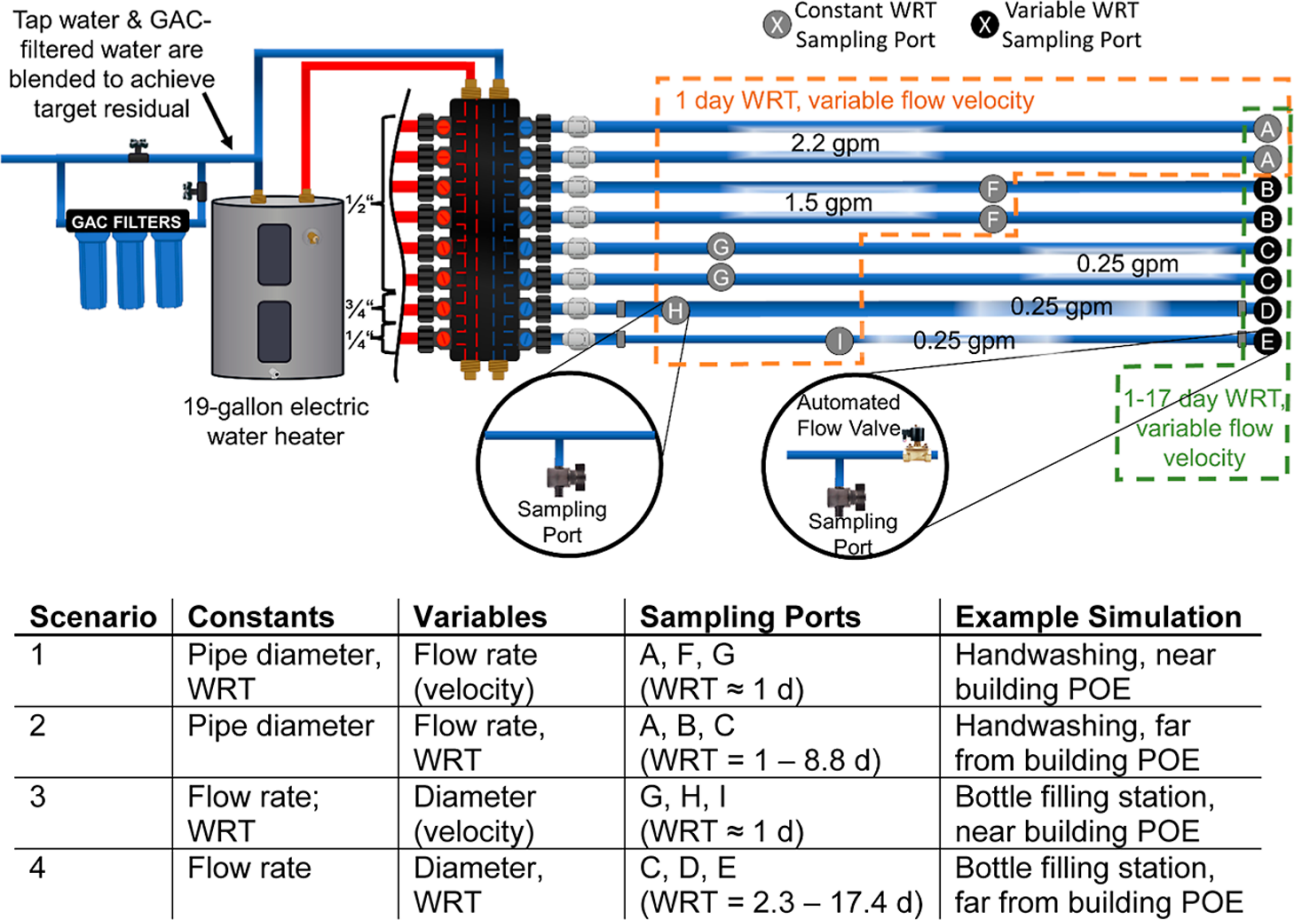
At-scale plumbing rig used in these experiments. Chloraminated tap water and GAC-filtered tap water were blended to achieve target influent chloramine residual. Check valves were installed at the beginning of each pipe to prevent backflow and cross-contamination. The “constant WRT” sampling ports were installed at the length along the pipe segments corresponding to the volume of one flush, represented by the gray ball valves (ports A, F, G, H, and I; locations not to scale; Table S1). “Variable WRT” sampling ports were installed at the end of each pipe, represented by the black ball valves (ports B, C, D, and E). Flow was automated using solenoid valves on timers installed at the end of each pipe. Flow velocities are listed in Table S2. POE: point-of-entry.

Extreme scenarios of hot and cold water lines were replicated using an at-scale building plumbing rig to systematically examine the influence of the flow rate and WRT on water quality (Figure 1; Table 1). Chloraminated tap water was flushed for 10 min to establish consistent influent water quality and then either filtered through three sequential granular activated carbon (GAC) filters to remove chloramine or mixed with different ratios of unfiltered (bypass) water to achieve the targeted influent chloramine residual. Target chloramine doses were 0.2 mg/L (EPA minimum leave the treatment plant) and 1 mg/L. The cold water flowed directly into cold water pipes or into a 19-gallon electric water heater where it was heated to a target temperature and distributed to hot water pipes. A manifold served 8 cold and 8 hot cross-linked polyethylene (PEX-B) water pipes with spring check valves to prevent cross-contamination between pipes. Each pipe was 134-ft long, simulating a long plumbing run to outlets within a home.

**Table 1:** Summary of Experimental Conditions and Expected Outcomes

phase	dates	NH_2_Cl as Cl_2_ entering cold pipes and water heater (mg/L)^b^	daily cold and hot flush^a^	cold water expected outcomes	hot water expected outcomes
I	February–April 2019	0.2 ± 0.17 (*n* = 12)	1×	highest levels of bacteria expected because of low flow rate and chloramine levels, especially at the lowest flow rate/highest WRT sampling port	at 37.8 ± 1.2 °C, most growth occurs in water heater due to 3-day WRT. Little to no additional growth is expected in hot pipes regardless of flow rate
II	June–August 2019	1.05 ± 0.19 (*n* = 24)	1×	bacterial levels established in the worst case scenario of phase I are expected to be reduced by influent NH_2_Cl	at 38.9 ± 0.8 °C, influent NH_2_Cl is consumed in the tank, resulting in some growth shifts from tank to distal lines. Low use and low flow rate cause lower NH_2_Cl and facilitate growth
III	September 2019	1.21 ± 0.06 (*n* = 2)^c^	cold: 10×; hot: 1×	further reductions in bacterial levels relative to phase II are expected as a result of higher levels of NH_2_Cl delivered more frequently	at 57.3 ± 0.4 °C, growth in the tank is minimized by elevated temperature and further shifts microbial growth into distal lines. The highest growth in pipes occurs at highest WRT
IV	October 2019	0.09 ± 0.07 (*n* = 2)^c^	cold: 10×; hot 1×	lower concentrations of bacteria expected compared to phase I due to dilution, but greater bacterial yield expected due to greater influx of nutrients	at 55.8 ± 1.9 °C, growth in the tank and pipes is similar to phase III

a 35 s flush duration.

b NH_2_Cl was measured as total chlorine.

c Phases III and IV are based on a limited sampling size and do not fully reflect the population of the experimental condition.

Two extreme consumer water use scenarios were examined: a fixed duration (e.g., restroom handwashing) or a fixed volume (e.g., water-bottle filling stations). In the case of a fixed duration, the volume of water drawn per event is proportional to the flow rate. For the fixed duration scenario, six 0.5 in. diameter pipes of equal length flushed at flow rates representative of commercially available aerator flow restrictors in duplicate, with slight variations in flow rate. In this scenario, the WRT was inversely related to the pipe flow rate (Figure 1, Scenario 2, sampling ports A–B–C) and pipe lengths were decreased to examine a constant WRT (Figure 1, scenario 1, sampling ports A–F–G). Sampling ports C–D–E were designed to assess situations where collecting a fixed volume is needed but pipe diameters differ (Figure 1, scenario 4). When the length of the plumbing was decreased for pipes of larger diameter in this scenario, the same WRT was achieved for all pipe diameters (Figure 1, scenario 3, sampling ports G–H–I). In scenarios 1 and 2, flow velocity (i.e., distance water travels over time) was controlled by the flow rate (i.e., volume of water moving over time), whereas in scenarios 3 and 4, flow velocity was controlled by the pipe diameter. To examine the impact of flow velocity in pipes at a fixed flow rate, pipes of 0.75″, 0.5″, and 0.25 in. diameter were flushed at 0.35 ± 0.02 gpm.

### Experimental Design

The rig was conditioned for 4 months to reach pseudosteady state conditions before the experiment began by flushing pipes 1×/day for 35 s, which is the median flow duration of residential hot water systems per use.^32^ The 35 s flush displaced the water volume in each pipe up to the 1 day WRT with influent water. Four experimental phases and associated expected outcomes are summarized (Table 1). Phase I tested a worst-case scenario for microbial growth, by minimizing influent chloramine, setting the water heater at 40 °C, and flushing all pipes 1×/day (Table S2). Phase II was the same as phase I, except cold water entering the cold pipes and the water heater tank carried a target residual of 1 mg/L Cl_2_. During phase III, target chloramine entering the system remained at 1 mg/L Cl_2_, but the hot water storage temperature was increased to 60 °C. Additionally, cold water was flushed 10×/day to determine the impacts of a more typical water use pattern. Phase IV was the same as phase III, except chloramine was again removed from the influent water via GAC filtration.

### Sampling Approach

Physicochemical parameters of the influent cold and hot water entering pipes, before and after routine water changes, were monitored to detect and account for any changes in the influent water quality over the duration of flushing events. Water samples collected from each sampling port included the first 250 mL and last 250 mL to quantify the change in each physicochemical parameter over the duration of the flush (Supporting Information 1). Sampling of each condition was conducted over 23 days during phase I (*n* = 12) and over 27 days during phase II (*n* = 24).

After both phases I and II, the rig was left to flush 1×/day for 17 days prior to collecting larger volume samples for quantitative polymerase chain reaction (qPCR). Phases III and IV were intended as briefer follow-up experiments to phases I and II and therefore were not sampled as extensively, and qPCR was not carried out.

Samples for microbial analysis were collected in sterile polypropylene bottles. When a disinfectant residual was present, 24 mg/L sodium thiosulfate was added to the sample. Samples collected for qPCR analysis included 1 L of cold and hot water entering pipe conditions and 0.5 L of first draw and last draw of all pipe conditions at each sample port. Sampling was conducted on three consecutive days at the end of each pipe sampling port (variable WRT), followed by three consecutive days at the midpipe-run sampling ports. At the end of both phases I and II, 15–40 cm^2^ (depending on pipe diameter) biofilm swabs were collected using sterile cotton tip applicators (Fisher Scientific, UK).

### Chloramine Decay Profiling

Chloramine profiles from the individual pipes were collected as a function of flushing (phase II only) and stagnation following phases II and III (the phases with chloramines) for each of the 0.5 in. pipe conditions. Methods and results of these data are presented in Figure S1.

### Analysis of Microbial Water Quality

Water samples were filter-concentrated, and DNA was extracted using the FastDNA Spin Kit (MP Biomedicals, Solon, OH). Gene markers for total bacteria, *Legionella* spp. and *Mycobacterium* spp., were enumerated by previously developed and validated qPCR assays (Table S4). Though qPCR detects both live and dead microorganisms, here, we consider growth as an increase in the gene markers within the at-scale plumbing rig relative to influent levels. Additional details regarding positive and negative controls, PCR inhibition, standard curve performance (efficiency and *R*^2^), and detection limits are presented in Supporting Information 2. *Legionella pneumophila* and *Mycobacterium avium* did not naturally colonize the system and were not detected when tested. Thus, the analysis of these organisms focused at the genus-level as an indication of OP growth potential. Total cell counts (TCC) were measured by flow cytometry, as described in Supporting Information 1.

### Data Analysis

A bulk water relative growth factor was defined as the total number of cells exiting the sampling port divided by the total number of cells entering the pipe. Although this parameter does not directly account for growth in the biofilm, it focuses on the levels of microbes experienced by the consumer at the tap (eq S1).

### Statistical Analysis

Statistical analyses were performed in RStudio (R version 3.4.3). Nonparametric Spearman rank correlations, two-sided paired and unpaired Wilcoxon Rank Sum, and Kruskal–Wallis tests were performed, as appropriate and detailed in the text. Significance was determined at *p* < 0.05.

## Results

Physicochemical trends for chloramine, ammonia, and temperature in the rig are first presented, followed by a more detailed examination of effects of rig operation on microbiology.

### Chloramine and Ammonia Levels in the Pipe Rig

During phase I, influent chloramine residual was reduced by the GAC filters from ∼3 to <0.22 mg/L as Cl_2_ (Table 2). The GAC filters also reduced total ammonia from ∼0.5 to <0.2 mg/L NH_3_–N. After a 3-day retention time in the 40 °C water heater and 1 day WRT in the cold water pipes, chloramine and total ammonia were always at or near the detection limit (0.04–0.06 mg/L Cl_2_ or NH_3_–N). The consumption of total ammonia was considered as a surrogate for nitrifying bacteria activity in the water heater tank.

**Table 2:** Average (± Standard Deviation) Influent Water Quality Conditions^a^

	cold water influent			hot water influent		
phase	Cl_2_ (mg/L)	NH_3_ (mg/L)	temp (°C)	Cl_2_ (mg/L)	NH_3_ (mg/L)	temp (°C)
I (*n* = 12)	0.22 ± 0.17	0.19 ± 0.12	18.3 ± 4.1	0.04 ± 0.04	0.06 ± 0.03	37.8 ± 1.2
II (*n* = 24)	1.05 ± 0.19	0.42 ± 0.09	24.8 ± 0.6	0.10 ± 0.08	0.34 ± 0.06	38.9 ± 0.8
III (*n* = 2)	1.21 ± 0.06	0.16 ± 0.21	27.0 ± 0.1	0.075 ± 0.02	0.35 ± 0.00	57.3 ± 0.4
IV (*n* = 2)	0.09 ± 0.07	0.18 ± 0.21	27.3 ± 0.4	0.035 ± 0.02	0.07 ± 0.05	55.8 ± 1.8

a Total organic carbon (total organic carbon) data are reported in Figures S2 & S3.

During phases II and III, influent cold water contained 1.05 and 1.21 mg/L as Cl_2_ and ammonia levels were 0.42 and 0.16 mg/L NH_3_–N, respectively. Chloramine and ammonia were consumed (<0.02 mg/L) in the cold water pipes after just 1 day WRT. During all phases of testing, the chloramine decreased to trace levels following the 3-day WRT in the water heater tank. However, during phase III, nitrification was suppressed by high temperature in the water heater tank, preventing ammonia losses in the tank, with ammonia subsequently entering the hot water pipe network being consumed within 1 day WRT.

During phase IV, with chloramine <0.1 mg/L as Cl_2_ and water heater set to 60 °C, the 0.18 mg/L NH_3_–N left in the influent water approached the detection limit after the 3-day WRT in the water heater tank or by the 1-day WRT in the cold water pipes. While temperatures >45 °C are thought to inactivate nitrifying bacteria,^33−35^ stratification at the bottom of the electric heater may have allowed nitrifier growth^13^ in the absence of chloramine disinfectant.

### Temperature Profiles in the Pipe Rig

The influent cold water experienced a seasonal increase from 18.1 to 27.3 °C as phases I to IV progressed (February to October 2019; Table 2), but the measured temperature at all sampling ports was consistently at room temperature (22.5–25.0 °C). Water in the stagnant hot water distribution pipes quickly decreased to room temperature, and temperatures were only slightly elevated at the hot water 1 day WRT ports (28.7–30.5 °C, data not shown) immediately after flushing.

### Bacterial Growth Profiles Across Pipe Rig

**Figure 2:**
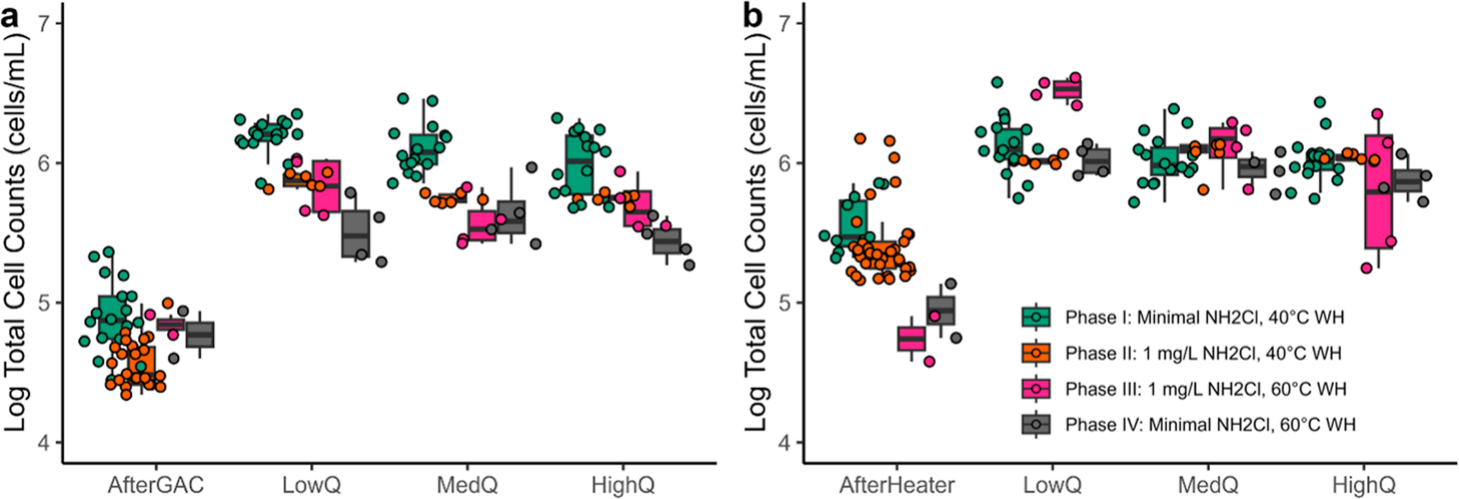
TCC in (a) cold water and (b) hot water at the point of entry to the manifold (after the GAC filters for cold and after the water heater for hot) and at the end of the distal pipes. Boxplots represent the 25th, 50th, and 75th percentile; whiskers represent 1.5 times the interquartile range. Individual data points are overlaid on top of the boxplots, and total sample numbers varied by phase. AfterGAC: cold water that entered the cold water pipe manifold and water heater tank; AfterHeater: hot water that entered the hot water pipe manifold; LowQ: low flow rate pipes (0.27–0.39 gpm, 0.32–2.47 fps, 0.61–11.92 day WRT); MedQ: medium flow pipes (1.37–1.49 gpm, 2.38–2.58 fps, 0.14–1.61 day WRT); HighQ: high flow pipes (1.99–2.2 gpm, 3.51–3.83 fps, 0.09–1.09 day WRT); WH: water heater set point.

The primary location of bacterial growth was governed by chloramine and the temperature. In the cold water, TCC in the distal pipes had a 10× relative growth compared to the water entering the pipes (“AfterGAC”; Figure 2a). When flushing frequency increased (phase I and II: 1×/day; phase III & IV: 10×/day), the TCC at cold water taps decreased about 50% (relative growth dropped to 5), likely due to insufficient WRT for growth and increased washout.

In the hot water system, TCC were highest in the water heater tank effluent during phases I and II, when the temperature set point was warm (40 °C) (Figure 2b). Slightly less cell counts were measured in the tank during phase II compared to phase I, presumably related to ∼1 mg/L chloramine entering the tank. Virtually no relative growth was observed in the tank effluent compared to the cold water influent entering the tank during phase III or IV, when the water heater was set to 60 °C.

Interestingly, regardless of the system, influent chloramine, or water heater temperature set point, the maximum cell density consistently was limited at an upper ceiling of ≈1000 cells/μL, in both hot and cold pipes. Thus, ≈1000 cells/μL appeared to represent the maximum total cell concentration possible for this system, potentially due to limitations in nutrients. Shorter WRTs (≤1 day) and elevated temperature or disinfectant essentially acted to limit the realization of this upper ceiling to the cell count.

### Cold Water Pipes

**Figure 3:**
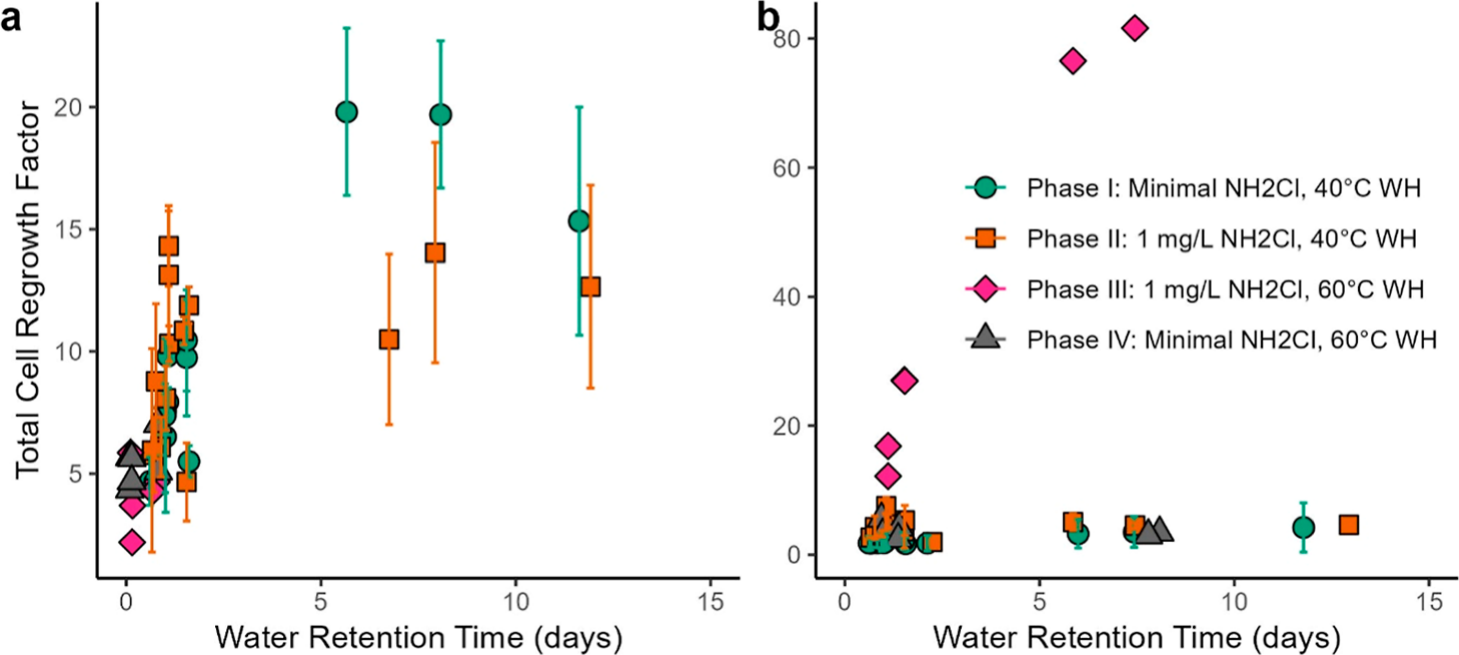
TCC relative growth factor as a function of water age in (a) cold and (b) hot water. The relative growth factor was calculated as total number of cells exiting a pipe divided by the total number of cells entering a pipe, as measured by flow cytometry (eq S1). Error bars represent standard deviation for *n* = 3 repeat measurements on each pipe. Duplicate pipes are plotted as individual data points, as flow rate varied slightly between conditions. High water ages correspond to the lowest water velocities in phases I and II. During phases III and IV, cold water was flushed 10×/day, eliminating water age as a covariate with flow velocity. WH: water heater set point.

In cold water pipes, the overarching factor affecting relative growth was WRT (Figure 3a). During phase I, with minimal influent chloramine, relative growth was 20 (i.e., 1.42 × 10^6^ cells/mL exiting the pipe was 20 times higher than the 6.65 × 10^4^ cells/mL entering the pipe) after 6.7 days WRT. At the highest WRT during phase I, the relative growth decreased to a level only 15 times more than influent total cells. We speculate that this lower relative growth is possibly due to variable release from the biofilm, nutrient limitation, or endogenous decay at very high WRT.

During phase II, growth in the cold water pipes plateaued at about a 2 day WRT and was limited to a relative growth factor of 14 compared to influent water. It appears that the higher influent chloramine slightly limited relative growth, even though chloramine was below detection at the sampling taps. It is also possible that there was nutrient limitation due to the overall low fresh water exchange frequency of 1×/day.^36^

We hypothesized that the higher flushing frequencies of cold water (10×/day) would have decreased relative growth due to more consistent delivery of the residual during phase III (with chloramine) or enhanced relative growth due to delivery of more nutrients in the absence of disinfectants in phase IV (without chloramine). However, the relative growth factor was limited to 5 for both phases, regardless of the flow rate. This is possibly due to a 10× lower WRT and bulk water dilution associated with more frequent water use and higher washout (Figure 3a).

### Hot Water Pipes

Compared to influent water, average TCC increased to 4.30 × 10^5^ cells/mL and 2.23 × 10^5^ cells/mL in the water heater tank during phases I and II, respectively (Unpaired Wilcox, *n* = 24, 48; *p*-value = < 0.001, <0.001), which translates to a relative growth of 5.5–6.5. The levels of TCC reached in the tank demonstrate a fairly consistent maximum relative growth potential due to a constraint such as a nutrient limitation. The decreased time frame to reach maximum growth in the water heater tank (<3 days) relative to the cold water pipes (∼6 days; Figure 3a) is consistent with a faster microbial growth rate at 40 °C. There was limited additional growth in the hot water pipes for all conditions relative to the water exiting the water heater. Because growth had already occurred in the water heater, there was <5 times increase in total cells in the pipes regardless of WRT (Figure 3b).

During phase III, relative growth in the water heater tank was very limited due to the elevated 60 °C set point and the presence of chloramine residual entering the heater. Cell counts only increased to 1.21 × 10^5^ cells/mL on average relative to the cold influent average of 5.9 × 10^4^ cells/mL (2.1 times increase). However, the amount of relative growth in pipes became a very strong function of WRT (Figure 3b). The relative growth factor was 82 at a ∼7.5-day WRT, which is 18× higher than the highest level observed in these same pipes for any phase I or II condition. This is consistent with the hypothesis that the influent cold water cells killed by elevated temperature and chloramine became bioavailable growth substrate for downstream bacteria in the hot water pipes.^37^ Despite the higher relative growth (i.e., number of cells exiting a pipe/number of cells entering a pipe), absolute cell growth (i.e., measured total cell count in cells/mL) was reduced at high flow taps in phase III compared to phases I and II (Figure 2b).

During phase IV, when chloramines were again removed from the influent water, growth once again occurred in the water heater tank, despite the elevated 60 °C setting, and the relative growth factor in downstream pipes was again limited to <10. Growth in water heater tanks could potentially occur in spite of elevated temperature set points as a result of stratification. A prior study documented that even at a 60 °C set point, 14% of the water heater tank volume can remain <46 °C, and the very bottom of the tank can be as low as 32 °C.^38^ Flow velocity did not affect relative growth beyond an inverse trend observed in hot water during phase III (Figure S4).

### QPCR Quantification of Growth in Phases I and II

#### Total Bacteria (16S rRNA Genes)

Trends in 16S rRNA (i.e., “total bacteria”) gene copy numbers were consistent with flow cytometry TCC, as detailed in Supporting Information 3.

#### *Legionella* spp. Growth

**Figure 4:**
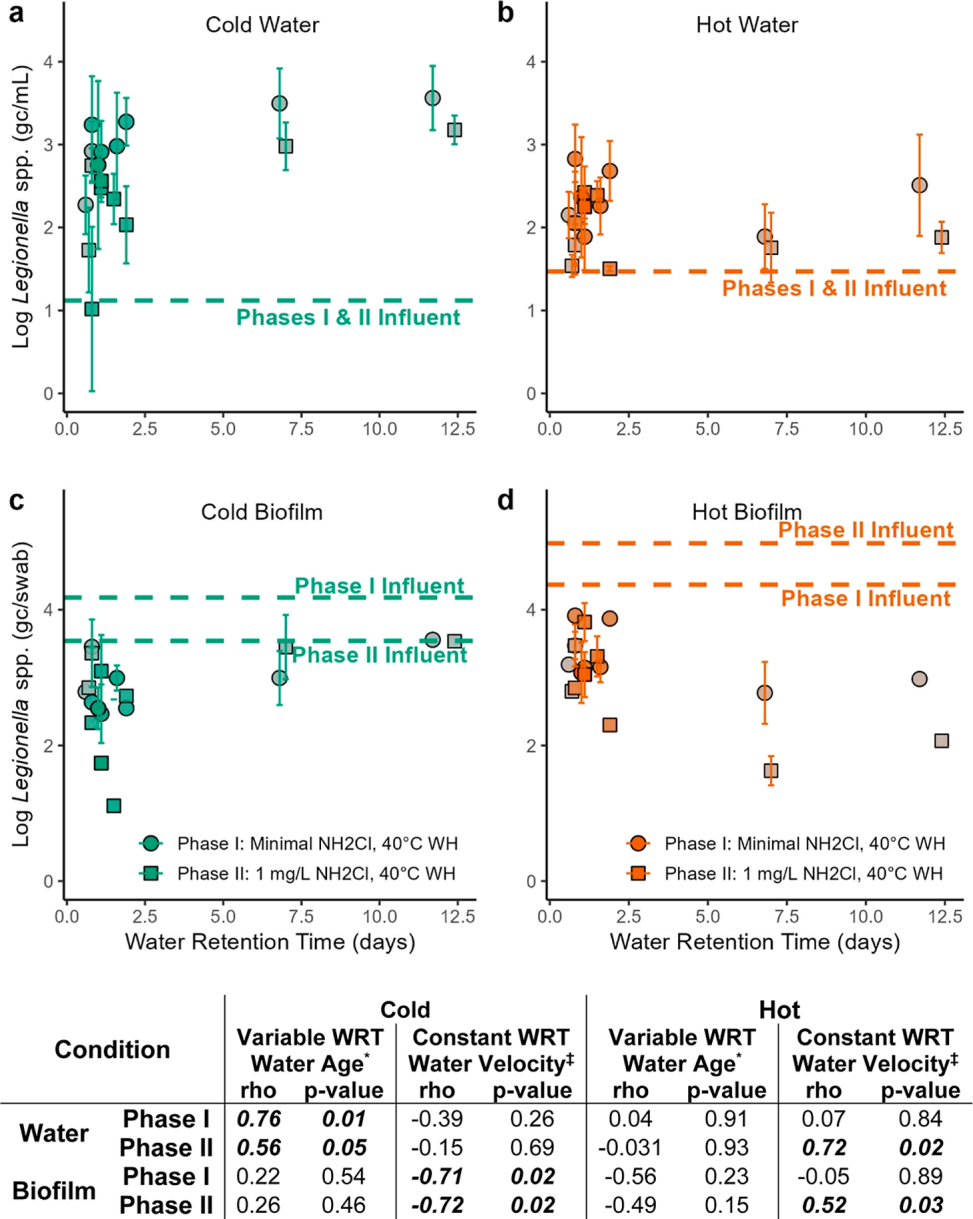
*Legionella* spp. gene copy numbers for (a) cold water, (b) hot water, (c) cold water biofilm, and (d) hot water biofilm. Horizontal lines represent influent levels of *Legionella* spp. gene copies in the pipes that supply the cold or hot manifold. Influent water gene numbers were not significantly different between phase I and II and are shown as an average of both phases. Biofilm influent levels trended differently between cold and hot water and are shown individually for each phase. Each line represents the average of two swabs (upstream and downstream of where the pipe was cut); thus, statistical comparisons are not possible. The intensity of the color of each data point (i.e., shading) is coded based on the flow velocity experienced by that sample. More intense/darker coloring represents higher flow velocity (max. = 3.81 fps), while less intense/lighter coloring represents lower flow velocities (min. = 0.33 fps). WH: water heater set point. Spearman rank correlation coefficients (rho) and *p*-values comparing gene copy numbers and WRT or water velocity are reported. *Correlations for water age were conducted on only the outlets with variable WRT (1–11.7 days for phase I; 1.1–12.4 days for phase II); ‡correlations for water velocity were conducted on only the outlets with constant WRT (WRT = 0.6–1.1 days).

In the cold water, *Legionella* spp. gene numbers increased at distal locations relative to the influent water (Kruskal–Wallis, *n* = 73, *p*-value = <0.001), and *Legionella* spp. gene numbers were a stronger function of WRT than velocity in each pipe (Figure 4). Across sampling ports capturing WRT = 1 to 11.7 days, *Legionella* spp. was positively correlated with WRT during phase I, with approximately 0.81 log more *Legionella* spp. at the highest WRT compared to 1-day WRT (Figure 4a; Spearman rank sum, *n* = 10, rho = 0.76, *p*-value = 0.011). At the constant WRT sampling ports (WRT ≈ 1 day), *Legionella* spp. gene numbers increased with water velocity during phase I (∼1.2 log increase from 0.33 to 2.7 fps; *n* = 24, rho = 0.72, *p*-value = 0.02), but not in phase II when chloramine was introduced.

*Legionella* spp. gene numbers in cold water biofilms did not increase in the distal outlets compared to the influent pipe (Figure 4c) and were more strongly impacted by flow velocity than water age (Figure 4c). *Legionella* spp. gene numbers in the biofilm positively trended with WRT, but the trend was not significant, whereas higher velocities were negatively correlated with *Legionella* spp. gene copy numbers in both phases (*n* = 24, rho = −0.71 to −0.72, *p*-value = 0.018–0.02).

During both phases in the hot water lines, *Legionella* spp. gene numbers increased by approximately 0.5 log in the water heater tank relative to the cold water influent (Figure 4a vs b; Unpaired Wilcox, *n* = 24, *p*-value = 0.034). Gene numbers further increased by 0.2–1.2 logs in the water phase in the distal lines but notably less than in the cold water pipes, and there were no significant trends with WRT in the water or biofilm. Hot water biofilm *Legionella* spp. gene numbers tended to decrease with WRT, but the result was not significant (Figure 4d). Similarly, the opposite trend to that in the cold water was observed in hot water and biofilms in phase II, wherein *Legionella* spp. gene numbers were positively correlated to water velocity (Figure 4b&d).

*Legionella* spp. gene numbers did not change in the influent cold or hot water between phases I and II (difference of 0.13–0.37 log gc/mL; Mann–Whitney *U* Test, *p*-value = 0.11–0.38), but there were diverging trends between hot and cold water in the biofilm. The lack of change in influent water *Legionella* spp. is likely due to increased seasonal growth during the summer months, when phase II was conducted, canceling out dilution from a lower fraction of GAC filtered water. In the distal outlets during phases I and II, *Legionella* spp. gene numbers increased by 0.41 log in cold biofilms but decreased by 0.62 log in hot water biofilms. While there were no differences between cold and hot water biofilms among the pipe conditions, cold water pipes consistently had higher *Legionella* spp. gene numbers than hot water at higher water ages.

#### *Mycobacterium* spp. Growth

**Figure 5:**
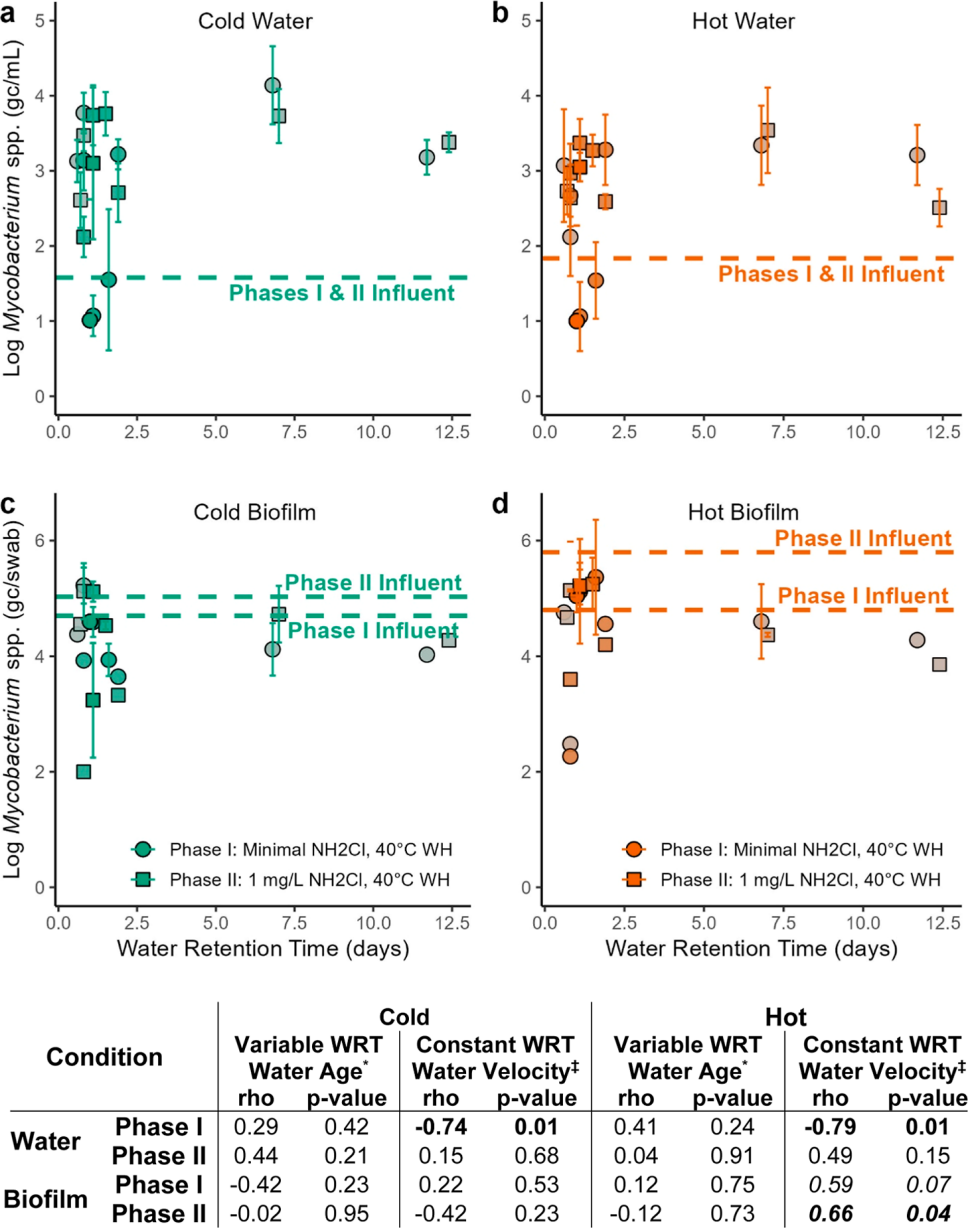
*Mycobacterium* spp. gene copy numbers for (a) cold water, (b) hot water, (c) cold water biofilm, and (d) hot water biofilm. Horizontal lines represent influent levels of *Mycobacterium* spp. gene copies in the pipes that supply the cold or hot manifold. Influent water gene numbers were not significantly different between phase I and II and are shown as an average of both phases. Biofilm influent levels trended differently between cold and hot water and are shown separately for each phase. Each line represents the average of two swabs (upstream and downstream of where the pipe was cut); thus, statistical comparisons were not possible. The intensity of the color of each data point (i.e., shading) is coded based on the flow velocity experienced by that sample. More intense/darker coloring represents higher flow velocity (max. = 3.81 fps), while less intense/lighter coloring represents lower flow velocities (min. = 0.33 fps). Spearman rank correlation coefficients (rho) and *p*-values between gene copy numbers and WRT or water velocity are reported. WH: water heater set point. *Correlations for water age were conducted on only the outlets with variable WRT (1–11.7 days for phase I; 1.1–12.4 days for phase II); ‡correlations for water velocity were conducted on only the outlets with constant WRT (WRT = 0.6–1.1 days).

*Mycobacterium* spp. gene numbers measured at constant WRT ports were negatively correlated with water velocity for both cold and hot water in phase I. In phase II, water velocity was positively correlated with hot biofilms (Figure 5). No other significant or consistent trends were noted for any other conditions. Cold and hot influent water did not change from phase I and II (Figure 5a&b), but *Mycobacterium* spp. gene numbers consistently increased in both cold and hot water biofilms during phase II when chloramines were elevated in the influent (Figure 5c&d). Cold water biofilms only increased by 0.2 logs, while hot water biofilms increased by >1.2 logs.

### Chloramine Decay Profiles in the Pipes

In the chloramine decay experiments, total chlorine in cold water pipes rose to influent levels only after flushing at least 4 pipe-volumes through each pipe (Figure S1a). Contrary to expectation, chloramine decayed faster in pipes with a higher water flow rate, with the high flow rate pipes losing 1 mg/L in just 5 h (Figure S1b). We speculate this is related to turbulence creating a more robust biofilm. High and median flow rate hot water pipes rose to the influent hot water chloramine residual level after flushing 2 pipe volumes. However, the low flow rate conditions did not rise to the influent chloramine rate even after flushing 5 pipe volumes (Figure S1c), suggesting there was a high level of chloramine demand as water flowed through these pipes or very nonuniform flow. Chloramine held in hot water pipes decayed approximately 2× faster than in the cold water pipes, and chloramine dropped below the detection limit after ∼10 h stagnation in all pipes (Figure S1d).

## Discussion

Our novel at-scale pipe rig compared multiple conditions in parallel to begin untangling the complex impacts of WRT, flow velocity, and flow rate on microbial growth, thereby revealing key vulnerabilities to pathogen growth potential under conditions representative of real-world building plumbing systems. The rig intersects a range of plumbing designs by replicating fixed duration (e.g., restroom handwashing) and fixed volume (e.g., water-bottle filling stations) use events considering scenarios for taps located near or far from the building point-of-entry. WRT directly impacts delivery of chemical and thermal disinfectant,^2,39^ and, correspondingly, was found to decrease overall growth potential. WRT also influences the duration pipes are within temperature ranges suitable for microbial growth.^40^ In the rig, rapid heat loss occurred during flushing, causing water temperature at hot distal outlets to be only slightly above room temperature.

This study provided new insight into the much discussed effects of pipe diameter and velocity on OP growth.^41−43^ For instance, one group reported that increasing flow velocity did not impact *M. avium* survival but did increase drinking water biofilm growth.^43^ In the circumstances studied herein, we found that the pipe diameter and flow rate had surprisingly little effect on microbial growth, except to the extent they influenced WRT and chloramine residuals. Chloramine decayed quickly in pipes after flushing events, especially in hot water lines and in low flow rate pipes, which have a higher WRT due to the large detention time in the water heater. There was significant total cell growth in the cold water pipes when no influent chloramine residual was present and when WRT was ≤3-days, but relatively little additional growth beyond 3-days. When chloramine was present in the water entering the pipes, there was reduced growth, even if the disinfectant residual was essentially nondetectable at the outlet. In hot water lines, the vast majority of growth occurred in the tank if it was 40 °C but very little growth occurred in the same tank at 60 °C.

There is disagreement in the literature regarding the extent to which shear stress and other hydraulic factors can affect growth and detachment of biofilms.^44−50^ In this study, water velocity was a covariate influencing WRT, but water velocity alone had no direct effect. Trends between *Legionella* spp. and velocity only occurred at constant WRT. Similarly, *Mycobacterium* spp. was correlated with velocity only at constant WRT (≤1 day). Studies have found that higher flow velocity can increase bacterial deposition from water to biofilms with adherence taking place within 1 day.^42,43^ Prior work conducted under continuous flow demonstrated that velocity has a major influence on fluxes of nutrients to biofilms,^30,51−53^ but such an effect did not occur herein, most likely, because the premise plumbing simulation was stagnant >99.6% of the time.

In evaluating optimal control of OPs, we demonstrate clear trade-offs between *Legionella* spp. and *Mycobacterium* spp. in response to chloramine dosing. Past research predicted that such a trade-off was based on observations of field sampling results which could have been confounded by the differences between the source waters tested.^54,55^ Employing an at-scale premise plumbing rig with a consistent water supply, this is the first controlled study to demonstrate that increasing chloramine could lead to lower *Legionella* spp. but higher *Mycobacterium* spp.

In hot water lines, the majority of *Legionella* spp. growth occurred in the water heater tank at 40 °C and no other prevalent trends were evident. *Mycobacterium* spp., on the other hand, significantly regrew in the water heater tank and exhibited substantial growth in low flow rate pipes at elevated WRTs. Thus, *Legionella* spp. mostly increased in the water heater tank when maintained at the ideal growth temperature range, and *Mycobacterium* spp. continued to increase with elevated WRT and low water velocities. These observations are consistent with current understanding of OP preferred growth niches.^37,56^*Legionella* did not significantly increase if water temperature was not suitable (e.g., in distal lines at ambient temperature or in the water heater tank when set to 60 °C). *Mycobacterium* preferred niches where it could compete with faster growing organisms (e.g., in low flow rate distal outlets with a high WRT or when chloramines killed competitors).

*Mycobacterium* spp. increased in the water heater when chloramine was present in phase II relative to phase I without chloramine. This is generally consistent with prior observations that *Mycobacterium* can persist and even increase in the presence of chloramine,^54,57−59^ although a mechanism for this has not been elucidated, and one study reported that chloramine did not impact Mycobacterium concentrations.^60^ This suggests that a native strain of *Mycobacterium* is present in the influent water, but whether they may be enriched on the GAC filter associated with the at-scale plumbing rig was not determined. Warmer temperatures and more nutrients in the water heater tank likely accounted for the >1 log higher growth that occurred in the hot compared to cold influent biofilm. Mechanistic studies are needed to better understand the circumstances under which *Mycobacterium* spp. can grow in chloraminated systems.

## Conclusions

This study employed a novel at-scale plumbing rig to enable parallel examination of multiple premise plumbing factors hypothesized to support the growth of OPs and other microbes. Conclusions of this study include:

- WRT was a key governing factor that controlled temperature and chloramine residual profiles.
- Pipe diameter and velocity played little role in suspended microbial growth in the extreme low flow frequency scenario investigated in this study.
- While chemical or thermal disinfection may delay microbial growth in premise plumbing, cell growth can restart once the disinfectant is absent and reach a maximum total cell count similar to conditions without disinfectant.
- *Legionella* spp. and *Mycobacterium* spp. had differing growth niches, with *Legionella* spp. gene copies being highest at optimal growth temperatures with minimal chloramine while *Mycobacterium* spp. was favored by chloramination.

## Supporting Information Available

Constant water age pipe length; phase I and II pipe conditions; physicochemical water quality analysis; chloramine decay profiling; assay information and references; DNA extraction and qPCR analyses; cell regrowth calculation; phase I TOC; phase II TOC; TCC relative growth factor as a function of water velocity; molecular quantification of growth in phases I and II; phase I 16s rRNA gene copy numbers; and phase II 16s rRNA gene copy numbers (PDF)
