# Supplementary material for: Untangling the Effects of Hydraulic Design on Opportunistic Pathogen Growth Potential with an at-Scale Plumbing Rig
Source: ACS ES T Water. 2025 Jan 3;5(2):738–48. doi: 10.1021/acsestwater.4c00812 (PMC11833858; doi:10.1021/acsestwater.4c00812)
Supplement: Supplementary file 1 — ew4c00812_si_001.pdf [file ew4c00812_si_001.pdf]

Supplementary information for

UNTANGLING THE EFFECTS OF HYDRAULIC DESIGN ON OPPORTUNISTIC PATHOGEN GROWTH POTENTIAL WITH AN AT-SCALE PLUMBING RIG

Sarah Busch[1§] and William J. Rhoads[1,2§], Tolulope O. Odimayomi[1], Amy Pruden[1], Marc A. Edwards[1*]

[1] Via Department of Civil and Environmental Engineering, Virginia Tech, Blacksburg, VA 24061, USA

[2] Current affiliation: Black and Veatch, 11401 Lamar Ave, Overland Park, KS 66211, USA

[§]Contributed equally to this work

*Email: edwardsm@vt.edu

Contents

UNTANGLING THE EFFECTS OF HYDRAULIC DESIGN ON OPPORTUNISTIC PATHOGEN GROWTH POTENTIAL WITH AN AT-SCALE PLUMBING RIG 1

Table S1. Constant Water Age Pipe Length 2

Table S2. Phase I & II Pipe Conditions 3

SI 1. Physicochemical Water Quality Analysis 3

Figure S1. Chloramine Decay Profiling 4

Table S4. Assay Information and References 6

SI 2. DNA Extraction and qPCR Analyses 7

Equation S1. Cell regrowth calculation 8

Figure S2. Phase I TOC 9

Figure S3. Phase II TOC 10

Figure S4. TCC Relative Growth Factor as a Function of Water Velocity 11

SI 3. Molecular Quantification of Growth in Phases I And II 12

Figure S5: Phase I 16s rRNA gene copy numbers 13

Figure S6. Phase II 16s rRNA gene copy numbers 14

References 15

Table S1. Constant Water Age Pipe Length

Target Flow Rate (gpm)	Nominal Pipe Diameter (in)	Constant Water Age Pipe Length (ft)*
0.25	0.25	57
0.25	0.5	15
1.5	0.5	92
2.2	0.5	134
0.25	0.75	8

*Example Constant WRT Pipe Length Calculation for 1/2" pipe flowing at 1.5 gpm:

$$V = Q * t = 1.5\ gpm * \frac{35\ sec}{60\ \frac{sec}{min}} = 0.875 \frac{gal}{flush} = 0.12\ ft^3$$

$$Where: V = volume\ (gal), Q = Flow\ Rate\ (gpm), t = time\ (s)$$

$$L = \frac{V}{A} = \frac{V}{\pi r^2} = \frac{0.12\ ft^3}{\pi * \left(\frac{0.485\ in * \frac{ft}{12\ in}}{2}\right)^2} = 92\ ft$$

$$Where: L = length\ of\ pipe\ (ft), V = volume\ (ft^3), A = cross - sectional\ area\ (ft^2),$$

$$r = radius\ of\ cross - sectional\ area$$

Table S2. Phase I & II Pipe Conditions

Measured conditions for the faucet rig during Phase I & II. Flow rate measurements were taken periodically between February-March 2019 for Phase I (n = 8) and II (n = 6). Average constant and variable water ages were based off of the measured flow rates for each pipe condition.

Number of Replicates	Target Flow Rate (gpm)	Measured Flow Rate (gpm)	Flow Velocity (fps)	Diam. (in)	Avg. Constant Water Age (days)	Avg. Variable Water Age (days)
1	0.25	0.32 ± 0.06	2.09 ± 0.38	0.25	0.83	2.3
2	0.25	0.33 ± 0.06	0.58 ± 0.11	0.5	0.76	8.8
2	1.5	1.43 ± 0.10	2.48 ± 0.17	0.5	1.1	1.5
2	2.2	2.19 ± 0.10	3.81 ± 0.18	0.5	1.0	1
1	0.25	0.37 ± 0.06	0.33 ± 0.06	0.75	0.61	17.4

SI 1. Physicochemical Water Quality Analysis

Total chlorine (Cl_2) and total ammonia (NH_3) were measured according to Standard Method 4500-Cl G and 8155-NH3 using a DR5000 spectrophotometer (HACH, Loveland, CO). The lower limits of detection (LLOD) where 0.02 mg/L for total chlorine and 0.01 mg/L for total ammonia. Total organic carbon (TOC) was measured by persulfate-ultraviolet detection using a Sievers Model 5300C according to Standard Method 5310 C with an LLOD of 4 µg/L. Total cells were measured with the BD Accuri C6 flow cytometer (LLOD = 1 cell/µL) with SYBR Green I dye.[1]

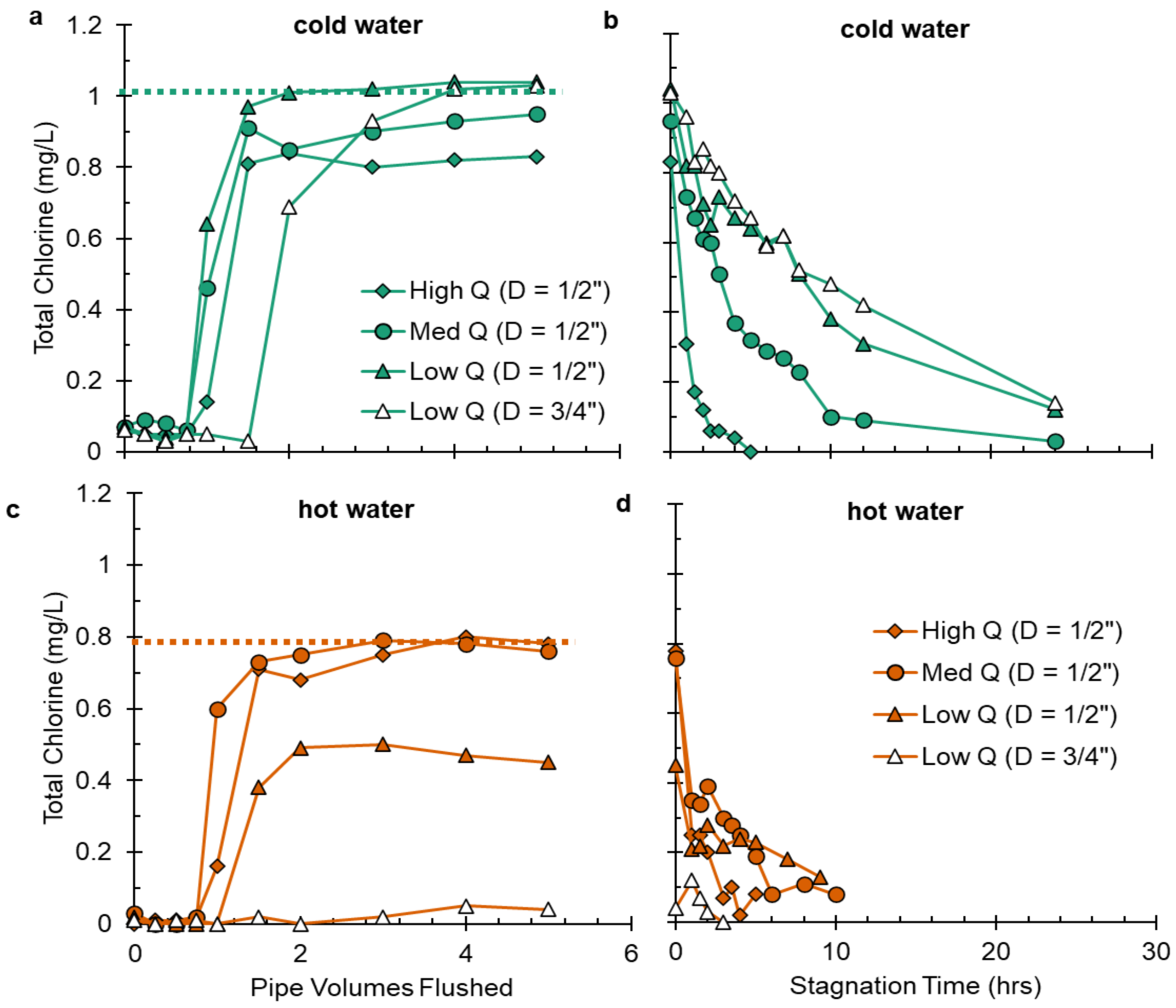

Figure S1. Chloramine Decay Profiling

Chloramine decay profiles measured as total chlorine in pipes with different flow rates as a function of pipe volume flushed and stagnation time in the pipe. Chloramine residuals for a) cold and c) hot water as a function of pipe volumes flushed; b) cold and d) hot water chloramine decay as a function of stagnation time in the pipe. Chloramine was established at the cold and hot water manifold (i.e., the influent to cold and hot water pipes) prior to flushing, indicated by the horizonal dashed lines. Pipe diameters are 0.5" unless stated otherwise in the legend. LowQ: low flow rate pipes (0.27-0.39 gpm); MedQ: medium flow pipes (1.37-1.49 gpm); HighQ: high flow pipes (1.99-2.2 gpm).

Chloramine profiles from the individual pipes were collected as a function of flushing (Phase II only) and stagnation following Phases II and III (the Phases with chloramines) for each of the 0.5" pipe conditions. To establish a disinfectant residual at the end of each pipe, five pipe volumes of water were flushed through the end of each pipe. Then, stagnation was initiated by closing the sampling ports at the end of the pipes, and small aliquots were then collected as a function of time, to assess the level of chloramine

present in the water held within each pipe. The entire water heater volume was flushed until the hot influent chloramine residual matched that of the cold water entering the experimental rig prior to flushing the hot water pipes, allowing delivery of chloramine levels representative of the water entering the rig to distal lines.

Table S4. Assay Information and References

Targeted organisms	Targeted genes	Sequences (5'-3')	Initial denaturation and enzyme activation	Denaturing / annealing / extension	Amplicon (bp)	Reference
Total Bacteria	16S rRNA	BACT1369F: CGGTGAATACGTTCYCGG<br>PROK: GGWTACCTTGTTACGACTT	98 ºC for 2 min	40 cycles of 98 ºC for 5 s and 55 ºC for 5 s	124	(Suzuki et al., 2000)[2]
Legionella spp. (qPCR)	23S rRNA	Leg23SF: CCCATGAAGCCCGTTGAA<br>Leg23SR: ACAATCAGCCAATTAGTACGAGTTAGC<br>Probe: HEX-TCCACACCTCGCCTATCAACGTCGTAGT	95 ºC for 2 min	40 cycles of 95 ºC for 5 s and 58.5 ºC for 10 s	92	(Nazarian et al., 2008)[3]
L. pneumophila (qPCR)	mip	LmipF: AAAGGCATGCAAGACGCTATG<br>LmipR: GAAACTTGTTAAGAACGTCTTTCATTTG<br>Probe: FAM-TGGCGCTCAATTGGCTTTAACCGA	95 ºC for 2 min	40 cycles of 95 ºC for 5 s and 60 ºC for 10 s	78	(Nazarian et al., 2008)[3]
Mycobacterium spp. (qPCR)	16S rRNA	110F: CCTGGGAAACTGGGTCTAAT<br>I571R: CGCACGCTCACAGTTA<br>H19R: FAM-TTTCACGAACAACGCGACAAACT	95 ºC for 2 min	45 cycles of 95 ºC for 5 s, 55 ºC for 15 s and 72 ºC for 10 s	462	(Radomski et al., 2010)[4]
M. avium (qPCR)	16S rRNA	MycavF: AGAGTTTGATCCTGGCTCAG<br>MycavR: ACCAGAAGACATGCGTCTTG	98 ºC for 2 min	40 cycles of 98 ºC for 5 s and 68 ºC for 18 s	180	(Wilton and Cousins D, 1992)[5]

Each 10 µL reaction contained 5 µl of 2× SsoFast Probes Supermix (Bio-Rad), 250 nM each primer, 93.75 nM probe, and 1 µl of DNA template. qPCR positive controls consisted of ATCC 33152 (*Legionella* spp.) and *M. avium* A5 (Beggs et al., 1995, courtesy of Dr. Joe Falkinham, III) that were cloned, amplified using M13 primers, and subjected to quantification by gel electrophoresis. Amplicon specificity was confirmed by Sangar sequencing and BLAST analysis (Wang et al., 2012). The effective LOQ and recovery efficiency corresponding to upstream sample processing (i.e., membrane filtration and DNA extraction), *Legionella* spp, and *Mycobacterium* spp., were spiked at defined concentrations in 500 ml of water and analyzed. Using this approach, LOQs were determined to be 32 and 170 CFU or cells/ml, respectively (Wang et al., 2012).

SI 2. DNA Extraction and qPCR Analyses

Water samples were filter-concentrated onto sterile 0.22-μm pore-size mixed cellulose ester filters (Millipore, Billerica, MA), which were fragmented and placed into DNA extraction tubes. For biofilm swabs, tips were aseptically transferred directly to DNA extraction tubes. DNA was extracted using the FastDNA Spin Kit according to manufacturer instructions (MP Biomedicals, Solon, OH). A DNA extraction negative control consisting of an unused filter was included with each batch of extraction.

Gene markers for total bacteria, *Legionella* spp. and *Mycobacterium* spp. were enumerated by previously developed and validated qPCR assays Table S4). Though qPCR detects both live and dead microorganisms, here we consider growth as an increase in the gene markers within the experiment apparatus relative to influent levels. DNA extracts, a negative template control (DNAse free water), and serial dilutions of positive controls were included in triplicate wells with each qPCR run. DNA extracts were diluted to minimize PCR inhibition, based on the results of a dilution curve conducted on a sub-set of samples. The quantification limit for qPCR was defined as the lowest standard that amplified consistently on each qPCR run resulting at an efficiency ≥80% and R^2≥0.98. The quantification limit for the 16S rRNA total bacteria assay was 500 gene copies/reaction but varied per run for other qPCR assays from 10-50 gene copies/reaction. Samples ≥ the quantification limit in at least two qPCR triplicate wells were considered quantifiable and were averaged in subsequent data analysis. Otherwise, results were recorded as below the quantification limit if there was amplification or non-detect if there was no amplification. All values are reported as log(gene copies/mL or swab + 1).

Equation S1. Cell regrowth calculation

Here, we define regrowth as the total number of total cells exiting a pipe over the course of each simulated water use minus the total number of cells entering the pipe

Cell Regrowth Factor =

Number of Cells Exiting Pipe / Number of Cells Entering Pipe

Number of Cells Entering Pipe =

Influent Total Cell Count (via flow cytometry, cells/mL) * Volume of Flush (mL),

Where, the Influent Total Cell Count was linearly interpolated between the Total Cell Count collected before and after the water changes occurred, to estimate the level of Total Cell Count entering the pipe for any given water change. And,

Volume of Flush =

Flow rate * Flush Duration

Number of Cells Exiting Pipe =

Total Cell Counts in the Last Draw Sample * Volume of Flush +

½ * (Total Cell Counts in the First Draw Sample - Total Cell Counts in the Last Draw Sample) * Volume of Flush

The ½ factor in the equation to calculate the number of cells exiting the pipe accounts for the change in total cell count between the first and last draw samples used for the linearly interpolation.

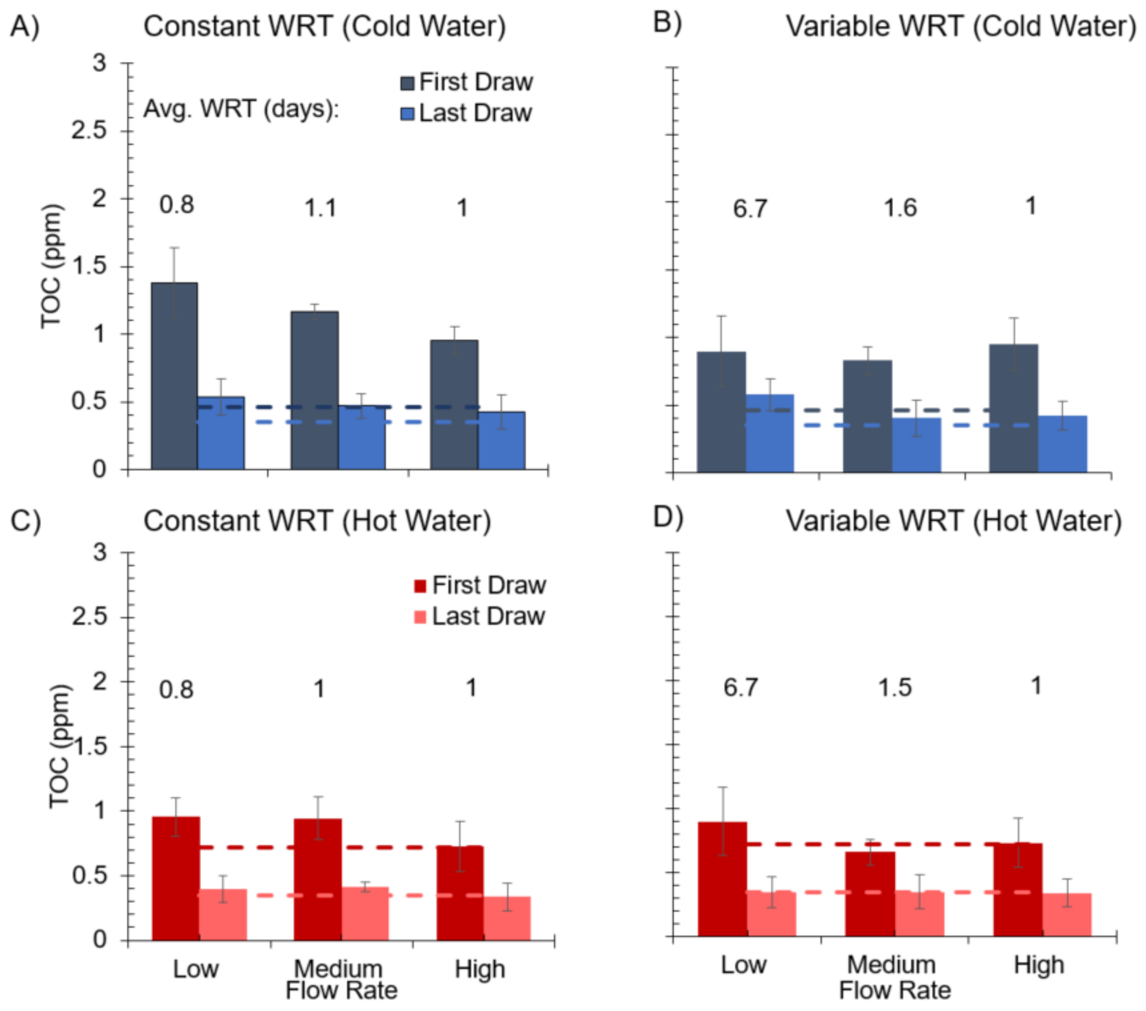

Figure S2. Phase I TOC

Total organic carbon (TOC) for A) cold-water flow rate at Constant WRT, B) cold-water flow rate at Variable WRT, C) hot-water flow rate at Constant WRT, and D) hot-water flow rate at Constant WRT. Error bars represent the standard deviation collected for respective hot and cold FD and LD samples (n =3).

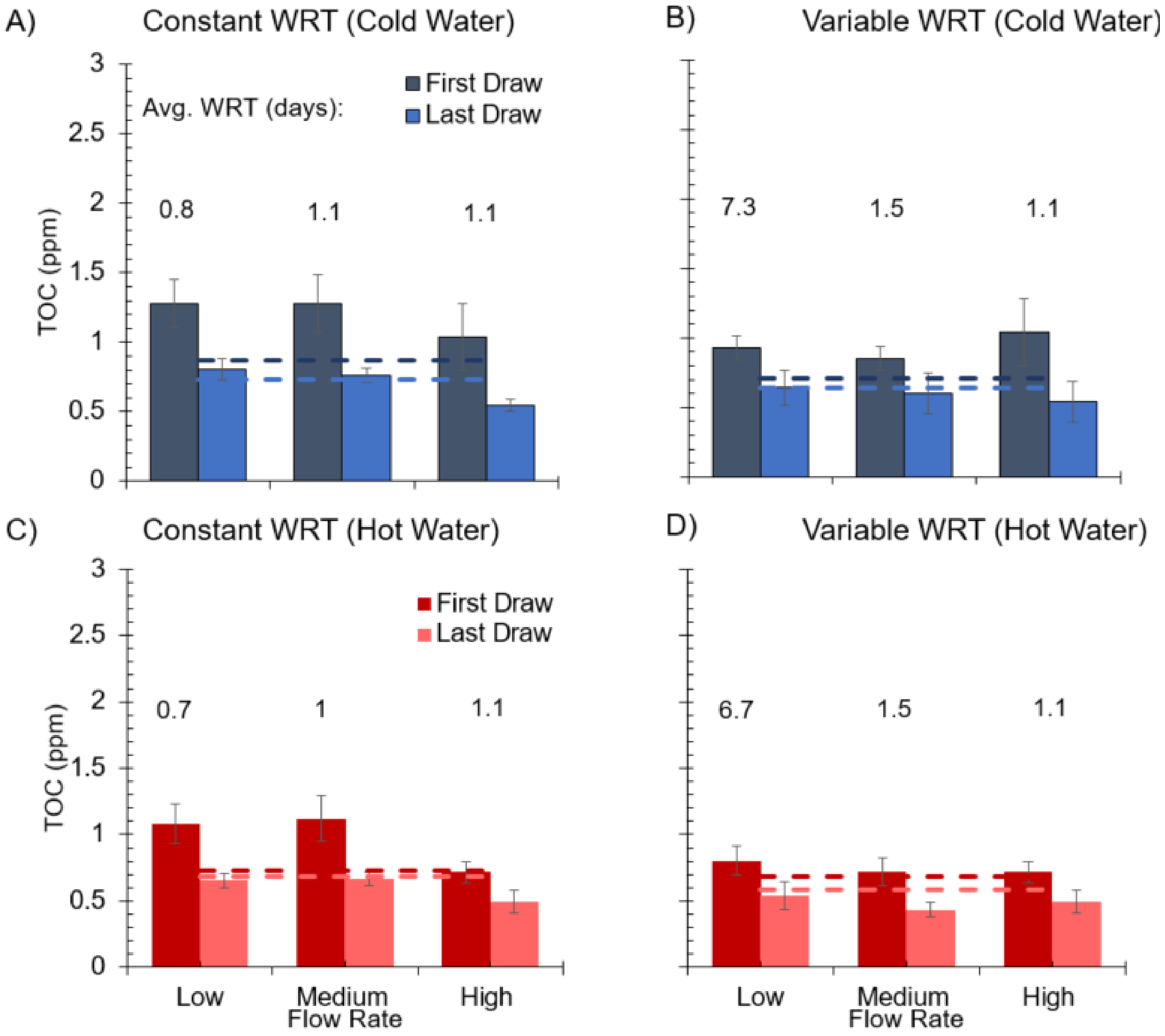

Figure S3. Phase II TOC

Total organic carbon (TOC) for A) cold-water flow rate at Constant WRT, B) cold-water flow rate at Variable WRT, C) hot-water flow rate at Constant WRT, and D) hot-water flow rate at Constant WRT. Error bars represent the standard deviation collected for respective hot and cold FD and LD samples (n =3).

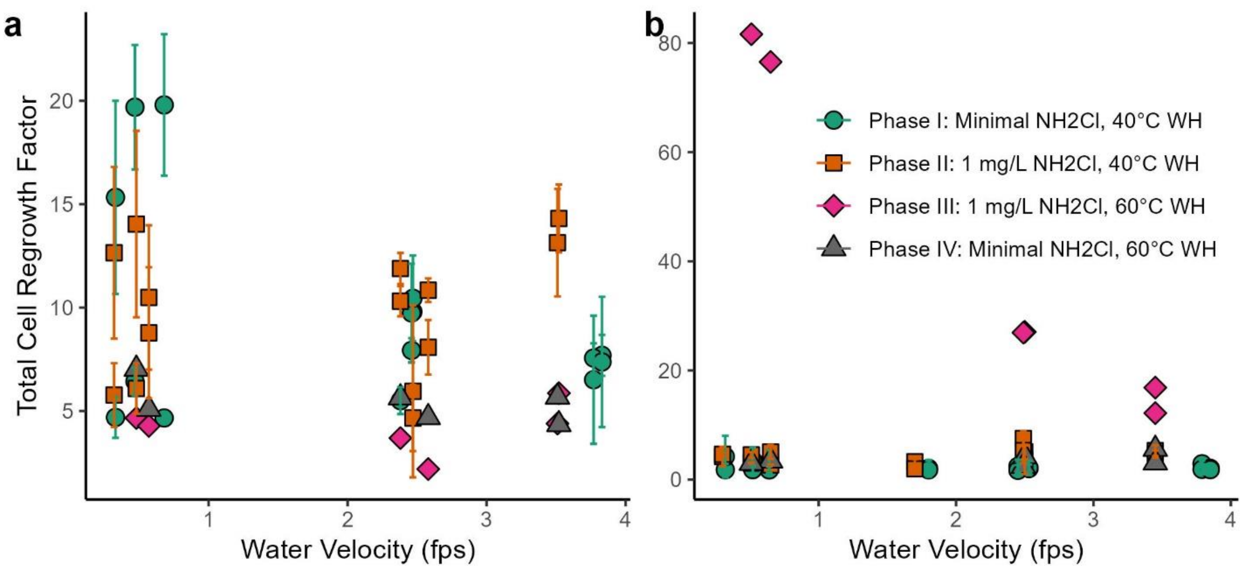

Figure S4. TCC Relative Growth Factor as a Function of Water Velocity

Total cell counts (TCC) relative growth factor as a function of water velocity in a) cold and b) hot water. The relative growth factor was calculated as total number of cells exiting a pipe divided by the total number of cells entering a pipe, as measured by flow cytometry (Equation S1). Error bars represent standard deviation for n=3 repeat measurements on each pipe. Duplicate pipes are plotted as individual data points, as flow rate varied slightly between conditions. High water ages correspond to the lowest water velocities in Phases I and II. During Phases III and IV, cold water was flushed 10×/day, eliminating water age as a co-variate with flow velocity. WH: water heater set point.

SI 3. Molecular Quantification of Growth in Phases I And II

Total Bacteria (16S rRNA genes). Trends in 16S rRNA (i.e., “total bacteria”) gene copy numbers were consistent with flow cytometry total cell counts. The 16S rRNA qPCR captures both live and dead cells; thus, it represents the maximum possible number of viable bacteria and has limited resolution for detecting immediate decreases in cell counts due to disinfection. During Phases I and II, there was approximately 0.2-0.4 log more total bacterial genes in hot water exiting the water heater tank relative to the cold water influent. With the addition of chloramine in Phase II, the levels of total bacterial genes decreased by 0.9 and 1.1 logs in the cold and hot water, respectively. Growth of total bacteria in the pipes followed a similar pattern to that of flow cytometry (Figure S5 & S6). For instance, during Phase I, bacterial growth in the water was a function of WRT and plateaued at approximately 20× higher than influent levels at WRT >5 days.

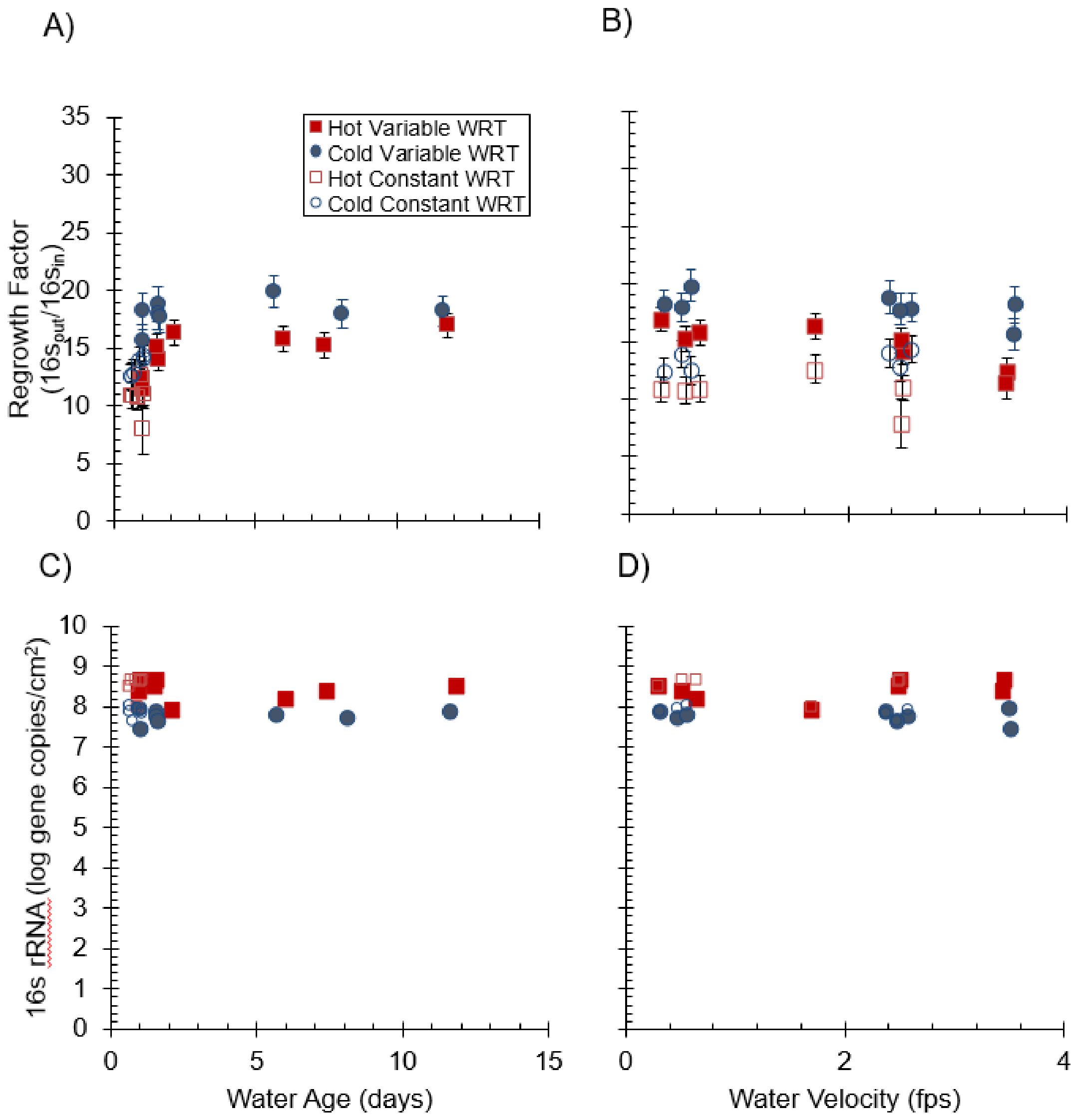

Figure S5: Phase I 16s rRNA gene copy numbers

A) regrowth as a function of water age, B) regrowth as a function of water velocity, C) biofilm as a function of water age, and D) biofilm as a function of water velocity. Error bars in A) and B) represent the standard deviation of n = 3 samples collected consecutively. No error bars are present for C) and D) because n = 1 samples were taken.

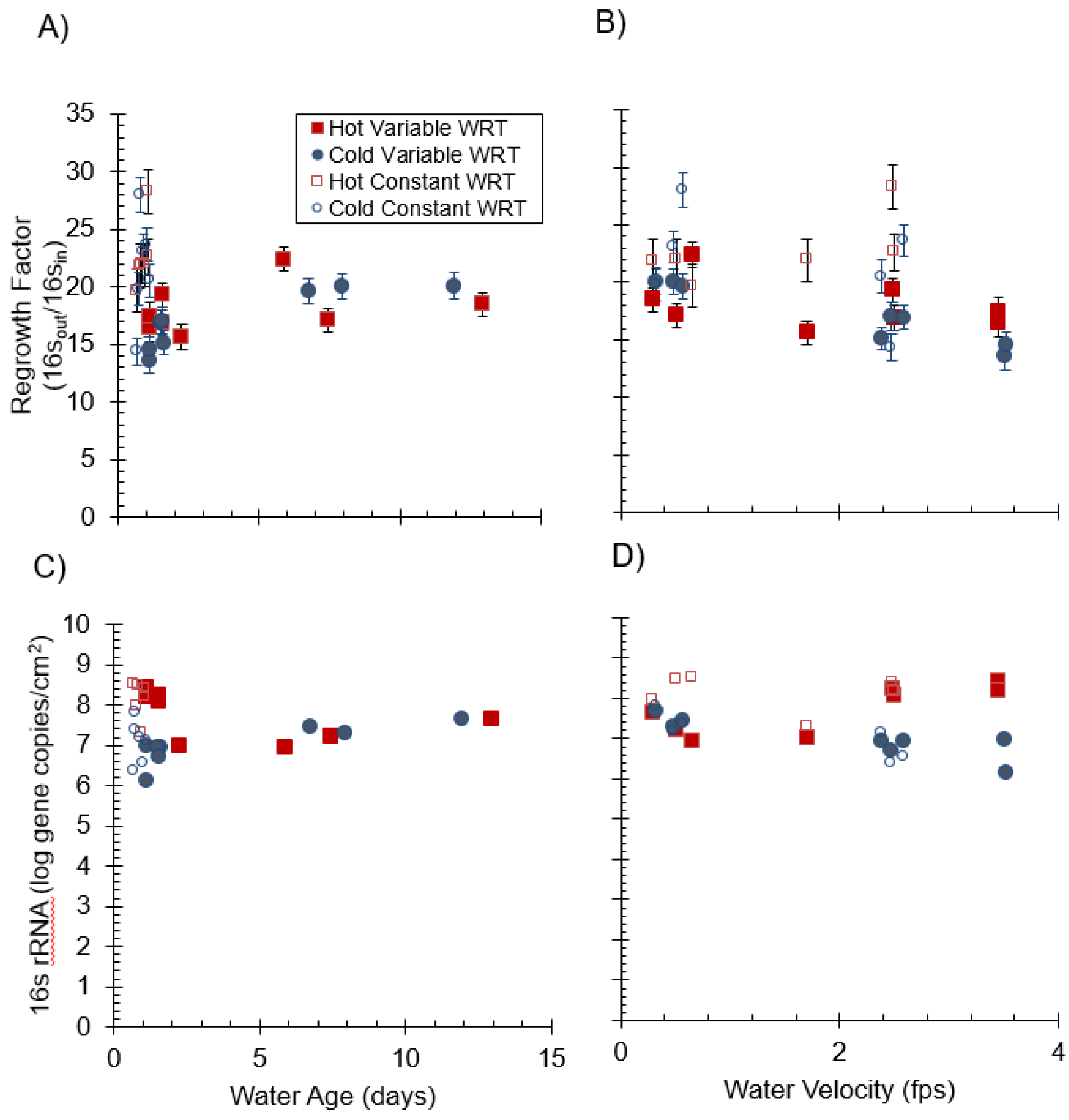

Figure S6. Phase II 16s rRNA gene copy numbers

A) regrowth as a function of water age, B) regrowth as a function of water velocity, C) biofilm as a function of water age, and D) biofilm as a function of water velocity. Error bars in A) and B) represent the standard deviation of n = 3 samples collected consecutively. No error bars are present for C) and D) because n = 1 samples were taken.

References

(1) Gatza, E.; Hammes, F.; Prest, E. *Assessing Water Quality with the BD Accuri™ C6 Flow Cytometer*; BD Biosciences: Ann Arbor, MI, 2013. https://www.umces.edu/sites/default/files/accuri-wp-assessing-water-quality.pdf.

(2) Suzuki, M. T.; Taylor, L. T.; DeLong, E. F. Quantitative Analysis of Small-Subunit rRNA Genes in Mixed Microbial Populations via 5′-Nuclease Assays. *Appl. Environ. Microbiol.* **2000**, 66 (11), 4605–4614. https://doi.org/10.1128/AEM.66.11.4605-4614.2000.

(3) Nazarian, E. J.; Bopp, D. J.; Saylors, A.; Limberger, R. J.; Musser, K. A. Design and Implementation of a Protocol for the Detection of Legionella in Clinical and Environmental Samples. *Diagn. Microbiol. Infect. Dis.* **2008**, 62 (2), 125–132. https://doi.org/10.1016/j.diagmicrobio.2008.05.004.

(4) Radomski, N.; Lucas, F. S.; Moilleron, R.; Cambau, E.; Haenn, S.; Moulin, L. Development of a Real-Time qPCR Method for Detection and Enumeration of Mycobacterium Spp. in Surface Water. *Appl. Environ. Microbiol.* **2010**, 76 (21), 7348–7351. https://doi.org/10.1128/AEM.00942-10.

(5) Wilton, S.; Cousins, D. Detection and Identification of Multiple Mycobacterial Pathogens by DNA Amplification in a Single Tube. *Genome Res.* **1992**, 1 (4), 269–273. https://doi.org/10.1101/gr.1.4.269.
